# Lateral migration of electrospun hydrogel nanofilaments in an oscillatory flow

**DOI:** 10.1371/journal.pone.0187815

**Published:** 2017-11-15

**Authors:** Sylwia Pawłowska, Paweł Nakielski, Filippo Pierini, Izabela K. Piechocka, Krzysztof Zembrzycki, Tomasz A. Kowalewski

**Affiliations:** Department of Biosystems and Soft Matter, Institute of Fundamental Technological Research, Polish Academy of Sciences, Warsaw, Poland; University at Buffalo - The State University of New York, UNITED STATES

## Abstract

The recent progress in bioengineering has created great interest in the dynamics and manipulation of long, deformable macromolecules interacting with fluid flow. We report experimental data on the cross-flow migration, bending, and buckling of extremely deformable hydrogel nanofilaments conveyed by an oscillatory flow into a microchannel. The changes in migration velocity and filament orientation are related to the flow velocity and the filament’s initial position, deformation, and length. The observed migration dynamics of hydrogel filaments qualitatively confirms the validity of the previously developed worm-like bead-chain hydrodynamic model. The experimental data collected may help to verify the role of hydrodynamic interactions in molecular simulations of long molecular chains dynamics.

## Introduction

The history of research on the lateral migration of particles in capillaries is a long one, inspired by the intriguing abnormality of blood flow characteristics [1–3]. Succeeding experimental and theoretical studies performed on the Poiseuille flow of solid [4–7] and liquid suspensions [8–11] have elucidated hydrodynamic mechanisms which are responsible for the characteristic cross-flow migration of the suspended particles. This migration mechanism appears to depend on the particle deformability, viscosity ratio, initial position, and flow rate. The importance of hydrodynamic interactions for polymer science surged after the pioneering works of Kratky & Porod [12], who identified the coupling of statistical thermodynamics with mechanical properties of chain-like models of macromolecules. Consequently, numerous theoretical studies appeared to predict the behavior of the fibers and filaments suspended in viscous fluids [13–22].

Early studies conducted with long polymer filaments indicated a unique migratory behavior determined, among other factors, by their effective stiffness stated in terms of the ratio of the persistence length *l*_*p*_ to the contour length *L*. For example, stiff filaments (*l*_*p*_ */ L* > 1) show a tendency to migrate toward the channel wall and to accumulate there as a result of the strong flow-induced alignment that decreases their diffusivity and promotes conformational stability [23–25]. In contrast, the migration of semiflexible filaments (*l*_*p*_ */ L* ~ 1) takes place preferably somewhere in between the channel wall and its center [23,26,27]. This is a consequence of an increased flexibility of the semiflexible chain compared to the stiff polymer that governs its unique U-shaped conformation and stabilizes its transport [28]. The migration of flexible filaments (*l*_*p*_ */ L* < 1), such as long DNA chains, usually proceeds in the direction of the microchannel centerline, where the polymer can sustain its equilibrium (coiled) conformation [29–34].

As pointed out by Graham [35] the long range hydrodynamic interactions may undergo substantial transformations under confinement, leading to significant changes of the molecular chains dynamics. The migration away from the wall is driven by the wall contribution to the hydrodynamic interactions. At the channel center there is no flow induced deformation of the chains, so they retain their equilibrium diffusivity. However, filaments diffusivity decreases as stretch increases therefore chains conveyed by fluid flow should migrate from the region of highest diffusivity toward regions of lower diffusivity, i.e. away from the channel center [35].

The *in situ* investigation of the hydrodynamic behavior of flexible polymer chains in microchannels is challenging due to the limited spatial-temporal resolution of standard fluorescent microscopes. It hampers the capture of the complexity of the filament chains configurational dynamics and fails to provide a direct insight into intramolecular interactions [36]. This is especially evident in the case of flexible polymers, such as DNA molecules, which show a significant increase in transverse bending fluctuations compared to semiflexible proteins (e.g. actin), resulting in a persistence length three orders of magnitude smaller.

Since the dynamics of highly flexible macromolecules is difficult to resolve properly by diffraction-limited fluorescent microscopy, the majority of the studies seeking to answer fundamental questions concerning the transport, configurational dynamics, and intramolecular interactions of flexible objects under hydrodynamic stress have been conducted *in silico* [15,16,36–39]. Only by significantly increasing the contour length of the polymer chain (e.g. by engineering a genomic (λ)DNA with a length of tens of micrometers) and using very narrow microchannels did it become possible to exceed shear stresses high enough to stretch coiled polymers and conduct hydrodynamic tests to validate computational results [14,29–33]. An increased length, however, adds additional complexity to configurational dynamics (such as knot formation) [40], while the increased confinement of microchannels may alter the dynamics and migration behavior of filaments under flow [35,36].

Here we report on the use of hydrogel nanofilaments as an alternative to long, highly deformable macromolecules to investigate their dynamics in an oscillatory microchannel flow. Hydrogels are known as water insoluble crosslinked polymers with water content up to 99%. Their high water content and structural similarities to the extracellular matrix create new opportunities in various bioengineering challenges. For the first time we were able to cast hydrogel material in form of nanofilaments [41], highly deformable objects in between nanoscale macromolecular chains and micro scale polymer fibers.

Our hydrogel nanofilaments were prepared by co-axial electrospinning, which ensures the formation of highly flexible structures with well-defined mechanical properties [41]. Such nanofilaments can thus be successfully used as a model system to systematically study the influence of their mechanical properties on hydrodynamic interactions and the resulting transport properties. Importantly, due to their simple wormlike mechanical structure, these electrospun nanofilaments make it possible to investigate the role of hydrodynamic forces in the deformation of flexible objects, without the need to take into account the complexity of molecular configurations. The use of these nanofilaments simplifies the mobility description, as we can ignore the influence of short-range molecular interactions, screening potentials, hydrophobicity, steric effects, and van der Waals interactions on their transport properties. In the following sections we show that the shape deformations and cross-stream migration of electrospun hydrogel nanofilaments in a microchannel flow are similar to those of long DNA biomolecules. We also show that these flexible hydrogel nanofilaments are capable of validating the essential assumptions of hydrodynamic bead-spring models [17–21].

## Materials and methods

### Materials

Poly-L-lactide-co-caprolactone (PLCL, 70% L-lactyde and 30% caprolactone) was purchased from Corbion Purac, the Netherlands. Chloroform (CHCl_3_) and N,N-dimethylformamide (DMF) were purchased from POCh, Poland. Bovine serum albumin conjugated with fluorescein (BSA-FITC), N,N isopropylacrylamide (NIPAAm), N,N’-methylene bisacrylamide (BIS-AAm), ammonium persulfate (APS), and N,N,N’,N’- tetramethylethylenediamine (TEMED) were purchased from Sigma Aldrich, Poland. Max Red Aqueous Fluorescent microsphere tracers dispersed in 1% (w/w) aqueous solution were purchased from Thermo Scientific™.

### Preparation of hydrogel nanofilaments

Hydrogels are known as water insoluble gels formed by a network of crosslinked polymer chains. Due to their high content of water, the manufacturing of fibrous nano-scale filaments from hydrogel material is a challenging task even when using a mold-based approach. The new approach based on the co-axial core-shell electrospinning technique (S1 File) [41] helped us overcome these difficulties and assemble hydrogel into nanofilaments with a diameter of around 200 nm. The selected hydrogel material was previously thoroughly characterized and marked as *EN1* [41]. Briefly, a hydrogel *core* solution was prepared from a mixture of NIPAAm/BIS-AAm/APS/TEMED/BSA-FITC with a mass ratio of NIPAAm/BIS-AAm (w/w) 37.5:1. The *shell* polymer was prepared by dissolving PLCL in a mixture of DMF and CHCl_3_. Both *core* and *shell* solutions were separately taken into two syringes having a common 20-gauge blunt needle tip. A 27-gauge needle was placed inside the 20-gauge needle to set the inner dimension for *core*-*shell* production (S2 File). These solutions were co-electrospun at a flow rate of 1.5 mL/h using a 15 kV voltage. Dispensed *core*-*shell* nanofibers with a typical diameter of about 1 μm were collected on a grounded drum rotating at 2000 rpm and placed 21 cm away from the needle tip. In order to extract the hydrogel *core* from its *core*-*shell* structure, the electrospun nanofibers were immersed into a 25% DMF-water solution and washed continuously till the fluorescent hydrogel nanofilaments were released completely. The nanofilaments obtained had a typical length varying from 1 μm to over 500 μm and a diameter in the range of 110 nm to 180 nm. The collected nanofilaments were left suspended in a DMF-water solution for several hours to stabilize their properties. To ensure the production of uniform hydrogel nanofilaments with coherent mechanical properties, constant values of all relevant parameters (e.g. solution composition, electrospinning, and hydrogel *core* extraction conditions) were maintained throughout all the experiments.

### Experimental setup

**Fig 1:**
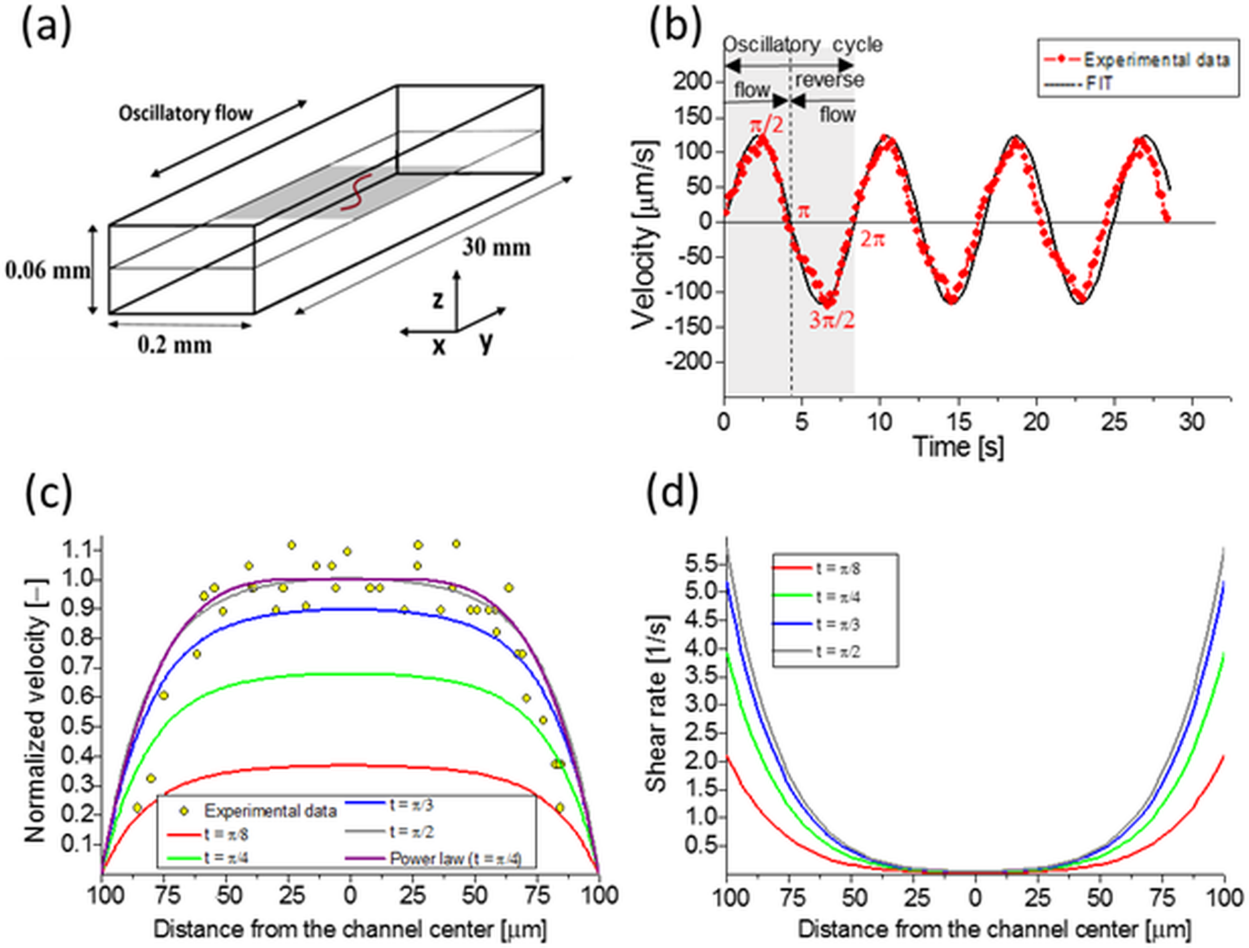
Characterization of flow field in an experimental microchannel. Schematic view of the flow configuration—(a); oscillatory flow waveform—(b); profiles of velocity- (c) and shear rate—(d) calculated for the plane of observations.

To investigate the hydrodynamic interactions and transport properties of nanofilaments, we placed them in the rectangular PDMS microchannel and subjected them to an oscillatory flow. Experiments were performed in a microfluidic channel with rectangular cross-section (200 μm x 60 μm) and 30 mm length (Fig 1A), fabricated from polydimethylsiloxane (PDMS) by soft lithography (S3 File). The channel was connected via capillary metal tubing to a home-built pulsate pump on one end, and to a syringe filled with a suspension of investigated particles (filaments or spherical tracers) on the other (S1 Fig). The oscillating velocity field was measured in the experimental cross-section of the channel by particle tracking method using dilute suspension of fluorescent tracers of 1 μm diameter (excitation 542 nm, emission 612 nm). Several experimental runs were performed at different flow rates and oscillation frequencies to evaluate basic characteristics of the flow pattern. Later on, these data have been used to validate analytical model of the flow velocity pattern and to calculate lateral migration of spherical particles subjected to the oscillatory flow.

Experimental runs with a suspension of fluorescently labeled (excitation 470 nm, emission 525 nm) hydrogel nanofilaments were initiated by manually injecting few of them through the tubing into the PDMS microchannel. Then they were subjected to an oscillatory shear flow by the pulsate pump. During experimental runs, the actual flow rate generated by the pump was verified by analyzing displacements of a few short (*L* ~ 1 μm) hydrogel fragments present in the solution. Results of these point measurements were inserted into described later theoretical model to calculate values of the local fluid flow velocity at its actual oscillation phase.

The maximum amplitude of the flow velocity (*V*_*max*_) used for the experiments was set between 0.05 and 0.95 mm/s and the forcing frequency varied between 0.12 and 0.64 Hz. The corresponding flow Reynolds number *Re* (= *V*_*max*_*Wρ*/η) based on the channel width *W* (S4 File), varied from 0.01 to 0.25. Suspended particles (nanofilaments and tracers) were observed through an inverted epifluorescence microscope (Leica AM TIRF MC) equipped with a 20x/0.40 NA microscopic lens, an appropriate set of filters, and a mercury lamp source (Leica EL6000) for fluorescence excitation. Their displacements were recorded using a high-gain EM-CCD camera (C9100-2, Hamamatsu) with a typical frame rate of 10 Hz. In total, from 500 to 2,000 individual images were recorded during a single experimental run.

Observations of particles were performed 15 mm from the inlet within the 500 μm-long and 200 μm-wide section of the horizontal central plane, located 30 μm below the top wall of the microchannel. The filaments or tracers located above or below the focal plane (focal plane thickness: 10 μm) were excluded from any further analysis. Moreover, filaments with a contour length that clearly changed during an experimental run were excluded from the analysis, as apparent changes of *L* indicated their out-of-plane shape deformations. All experiments were performed at the stabilized temperature of 302K, using the environmental chamber of the microscope. The working liquid density *ρ* and dynamic viscosity *η* measured at that temperature were 984 kg/m^3^ and 7.5 10^−4^ kg m/s, respectively. Experimental runs with polystyrene tracers were conducted in 10 mM KCl aqueous solution to avoid agglomeration. At all experimental runs concentration of particles (tracers and filaments) was below 2^.^10^-6^ w/w, to avoid any effects of inter-particle interactions.

### Simulations of the flow velocity field

The obvious limitations of the creeping flow models describing oscillatory flow in a rectangular channel forced us to perform numerical simulations. It should answer questions concerning the channel entry disturbances, possible hydrodynamic nonlinearities, and the reverse flow close to the channel walls. Hence, the finite element numerical platform (COMSOL Multiphysics Release 4.3b, COMSOL, Inc., Burlington, Massachusetts, United States) was used to simulate a time dependent three-dimensional velocity field of the water pumped into a rectangular microchannel. A fully coupled solver of Navier-Stokes equations with backward differentiation formulas for adaptive time stepping was used. The experimental microchannel was modeled as a computational domain consisting of 735,000 hexahedral elements. The dimensions of the domain were set to match the exact *x*-*z* dimensions of the experimental channel (200 μm × 60 μm), while the length of the domain was set to 30 mm. A time-dependent parabolic velocity profile was set at the inlet boundary according to the formula:

$$V(x,t)=V_{max}(1-{\underline{x}}^{2})sin(\omega t)$$ (1)

where *V*_*max*_ is the absolute maximum flow velocity in the center of the microchannel, *x* is the relative distance from the channel axis, *ω* is the angular frequency of oscillations, and *t* is the time. The channel inlet and outlet were set open to enable the flow-through fluid motion, according to the forcing time step. No-slip conditions were applied on the remaining microchannel boundaries.

### Evaluation of hydrogel nanofilament properties

The unique mechanical properties of hydrogels are of major significance in numerous biomedical applications. However, their mechanistic understanding is quite complex and difficult to characterize using the simple terms of elastic or plastic materials. Several modes of deformation can be identified, even within the limits of small displacements [42,43]. A deeper analysis usually entails the need to define new constitutive relationships [44], something which is beyond the scope of this report. Taking these constraints into account, we limit our description of the filament material characteristics to just two quantifiers: the bending Young modulus *E*, and the stretching Young modulus *E*_*x*_. The first characteristic is directly related to filament persistence length *l*_*p*_, and, in principle, it describes its deformational characteristics under thermal fluctuations. Its value has been established by analyzing several hundred microscopic images of hydrogel filaments suspended in the working fluid. The evaluation procedure was described in detail in our previous paper [41].

Briefly, the projected contour shape of the filament was fitted to a smooth curve. Then the variation of the tangent vector along the fitted contour was evaluated using an appropriate Matlab software program [41,45]. The data obtained were then used for the statistical analysis of spontaneous filament deformations. The *cosine correlation* method, applied directly, provides the bending stiffness κ of the sample, which is measured in terms of thermal energy *k*_*B*_*T* of its shape fluctuation characteristic *l*_*p*_:

$$\kappa =k_{B}Tl_{p}$$ (2)

By assuming a cylindrical shape of the filament, its bending Young modulus was obtained from the classical formulation for the second moment of inertia *I = π d*^*4*^*/64* of a rod with diameter *d*:

$$E=64\;\kappa /\pi d^{4}$$ (3)

The selected hydrogel material *(EN1)* was thoroughly investigated in the previous paper [41] using the above-mentioned statistical analysis, the direct bending of sample filament in shear flow, and the AFM indentation method. As this material appeared to be too soft for nanoindentation, we reviewed the data obtained from the first two methods and–by selecting the most reliable measurements–estimated that the value of the bending Young modulus *E* of our hydrogel filaments was equal to *2 kPa*.

The extensional Young modulus *E*_*x*_ could not be measured directly for our hydrogel filament samples due to their high fragility. Thus, a separate experiment was performed by extending 18 mm long hydrogel beam with a diameter of 4 mm and evaluating its stress-strain characteristics. By assuming the possibility to scale this data down to the nanofilament geometry, the estimated extensional Young modulus of our hydrogel filament appears to be one order of magnitude higher than the bending rigidity, i.e. *E*_*x*_ *= 20 kPa*.

Both mechanical values, i.e. bending modulus *E* and extensional modulus *E*_*x*_, were used to evaluate the relative flexural stiffness *A (*= *Ed*/32*ηV*_*max*_)) and the relative extensional (Hookean) stiffness *K* *(*= *E*_*x*_*d*^2^/4*LηV*_max_)*)* of our hydrogel filaments (S4 File), using a formulation proposed in previous studies [18–20].

The proper estimation of the hydrogel filament geometry, i.e. their contour length *L* and diameter *d*, plays an important role in analyzing their dynamics. The contour length was evaluated directly using microscopic images and the software already used for the persistence length evaluation. The long sequences of filament images taken in the channel before the oscillating flow started make it possible to obtain their average contour length along with the corresponding persistence length. The error of the *L* estimation depends mostly on out-of-plane filament deformation, and when its variations exceeded 10%, such data were excluded from further analysis. The persistence length of the filament obtained from these measurements was used to calculate its effective diameter *d*, according to the above-mentioned formula (3). This procedure appeared to be much more accurate than the analysis of microscopic images.

### Data analysis

Numerous geometrical characteristics can be defined to quantify the character of the complex deformations undergone by filaments during several periods of oscillating flow. Here, we limit our analysis to the buckling, inclination, and end-to-end distance only. The first parameter characterizes the degree of buckling or looping measured as the ratio of the diameter of the smallest circle enclosing the filament image to its contour length. Thus its value is always below one. The second parameter characterizes the inclination angle of the long axis of the ellipse encircling the filament. Its value changes from –π/2 for objects oriented into the channel inlet to +π/2 for those oppositely oriented. The end-to-end distance related to the filament contour length is selected as the third shape characteristic. It depicts the bending degree for relatively short filaments.

**Fig 2:**
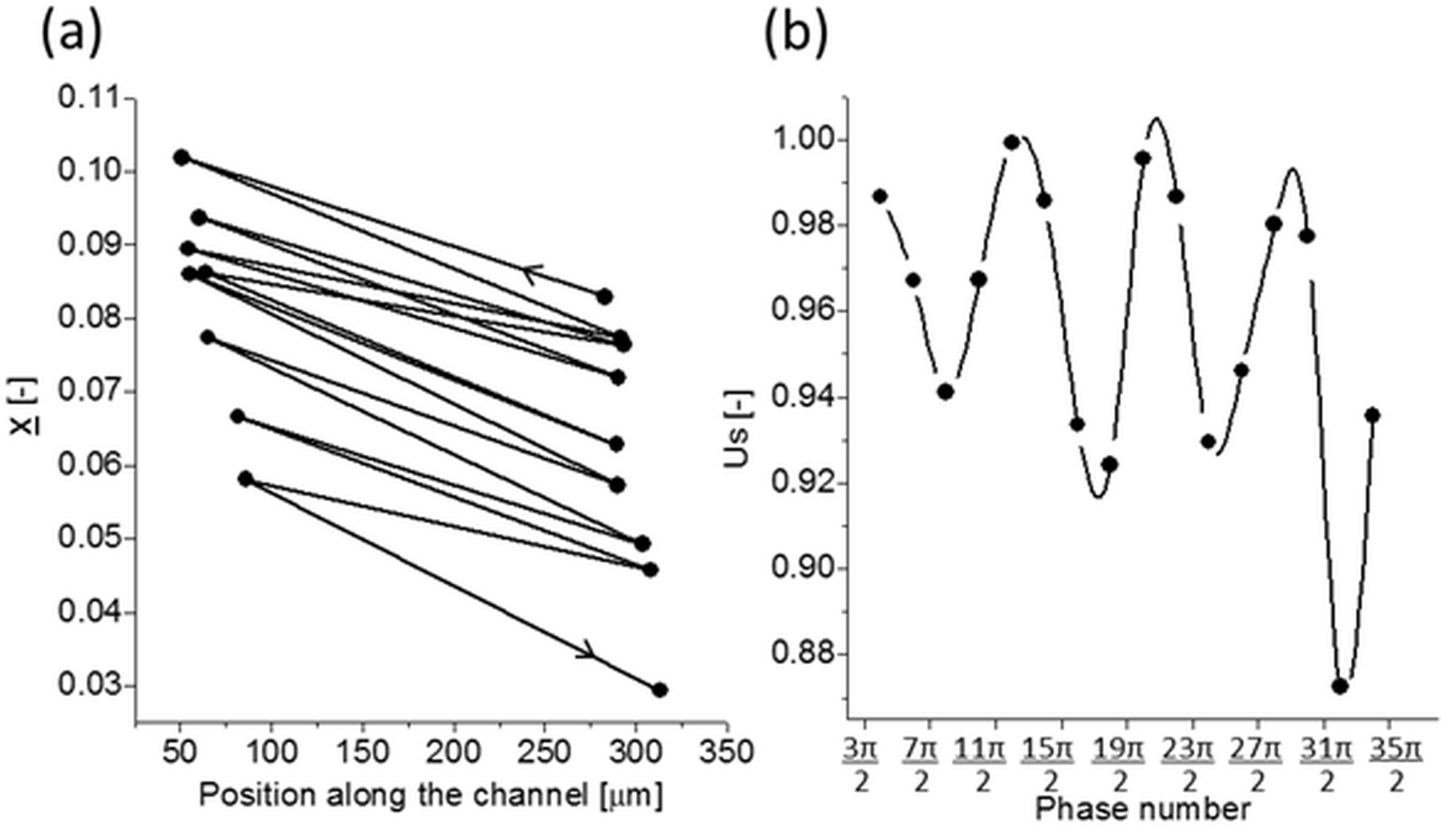
An example of U-shaped hydrogel nanofilament in the oscillatory flow, *d* = 181 nm, *L* = 32 μm, *V*_*max*_ = 282 μm/s, *U*_*r*_ *=* 10^−3^. (a)–lateral migration path of the nanofilament into the channel axis, center of mass position at each zero-crossing of the flow oscillation (n * π, n = 1,2,3.); (b)–relative longitudinal slip velocity *U*_*s*_ of the filament observed for each maxima of the oscillating flow (n * π/2, n = 1,3,5.). Remarkable out of phase pattern due to the filament deformations.

In addition to the periodic changes in shape and position, in most of the investigated cases the center of mass of the observed filaments migrates across the channel (comp. Fig 2A). Thus the absolute value of the migration velocity *U*_*r*_ is defined as the ratio of the averaged over total observation time absolute lateral filament velocity related to the maximum fluid flow velocity (*V*_*max*_). The longitudinal velocity of the filaments slightly lags behind the fluid flow velocity, its relative value is marked as a *slip* velocity *U*_*s*_. It is worth to note that observed variation of the *slip* velocity is out of phase with the flow oscillations due to the changes of filament shape and orientation (comp. Fig 2B).

## Results

### Channel flow

The oscillating flow configuration resembles the *in vivo* conditions where bio-objects are transported by inter-tissue time-dependent flow field fluctuations [46–49]. At the same time, the oscillatory flow made it possible for us to visualize the nanofilament dynamics in one detection regime with relatively long recording times. The maximum amplitude of imposed flow oscillations remained constant throughout the entire observation period, both in the first and in the second half of the cycle, where the reverse flow is applied (Fig 1B). The flow velocity reached its maximum absolute value at ± *nπ*/2, where *n* = 1, 3, 5, etc. Changes between cycles are smooth and without any phase lags. Our numerical simulations indicated the possibility to assume that the flow we impose is in the quasi-steady, creeping flow regime. The calculated hydrodynamic entrance-length is around 100 μm, short enough to neglect its effects in the observation area. Thus the fluid flow profile in the vicinity of the observation plane may be simply parametrized by time-dependent blunted parabolic function, approximated by a higher order polynomial. Fig 1C shows the experimental points obtained from displacements of the tracers, the velocity profile extracted from numerical simulations, and the fitted parabolic function with the exponent 4.5. Later on, this function was used to evaluate flow velocity and shear rate near the filaments (Fig 1C and 1D). The velocity profile across depth (*z*-dimension) of the microchannel is nearly parabolic, and the corresponding shear rates are much higher than in the *x*-*y* direction. This implies higher migration rates of suspended particles toward the observation plane. It is worth noting that the rectangular cross-section of the channel is responsible for characteristic dead zones in its four corners. At high enough flow rates, their existence may modify the behavior of the particles conveyed there and force their recirculation.

**Fig 3:**
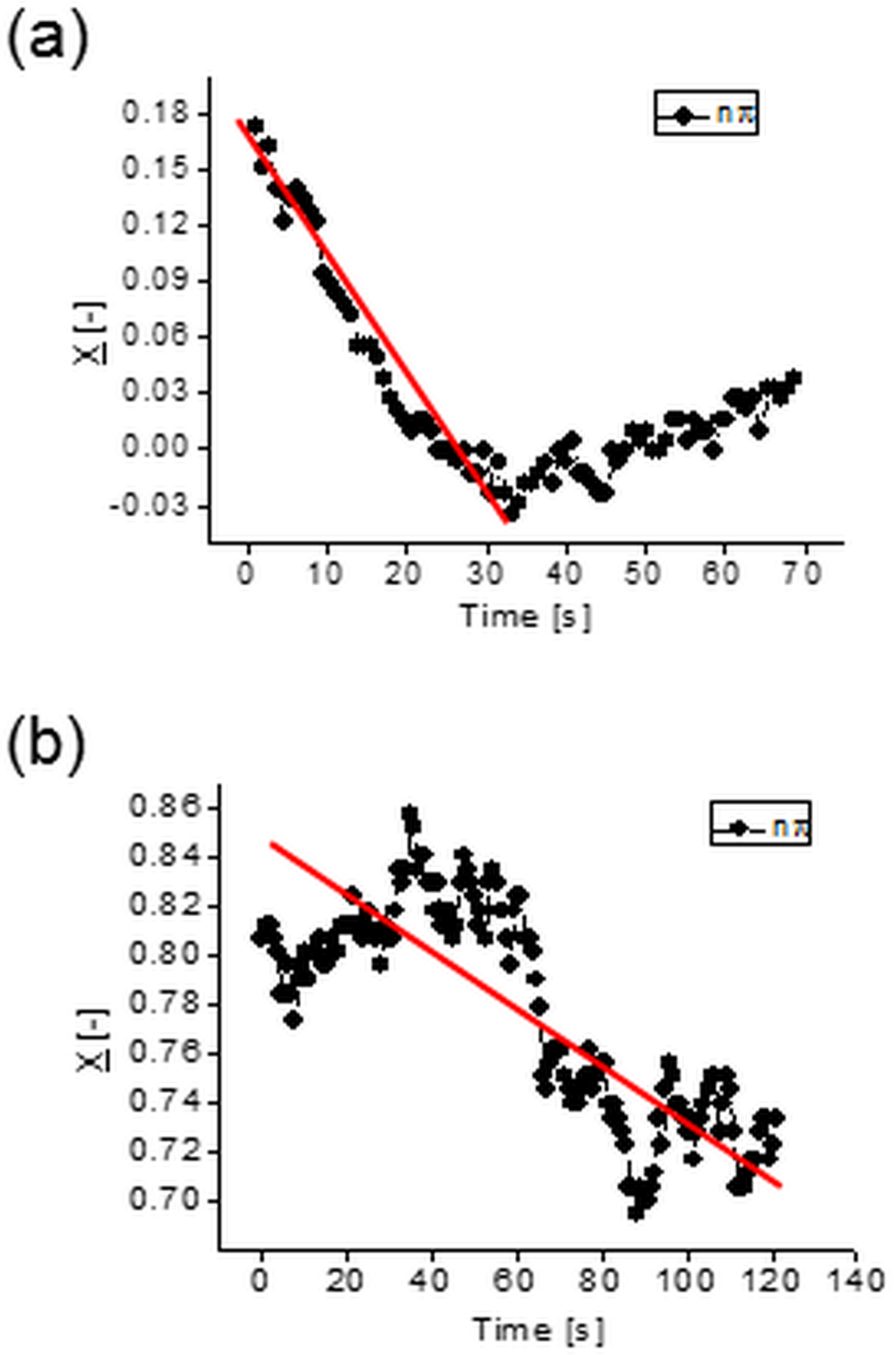
Lateral migration of *1*μ*m* spherical tracer in the oscillatory channel flow, particle position measured at zero-crossing of each oscillation period. (a)—*V*_*max*_ *= 259* μ*m/s; Re = 0*.*063; ω = 5*.*9*, slope of the line indicates relative migration velocity *U*_*r*_ *= 2*.*1 10*^*−3*^; (b)—*V*_*max*_ *= 119* μ*m/s*, *Re = 0*.*046; ω = 5*.*9; U*_*r*_ *= 0*.*6 10*^*−3*^.

Experiments performed with spherical tracers for the velocity field evaluation were extended to observe long term behavior of tracers after hundreds of flow oscillation periods. In fact, it was found that despite relatively low flow Reynolds number (*Re < 0*.*1*) their lateral migration across the channel is well pronounced. In most of the cases the spherical tracers were observed to migrate toward the channel center (Fig 3A and 3B) with the average velocity ranging from 0.1 μm/s to 7 μm/s. Observed irregular variations of their position indicate concerted action of effectively strong Brownian fluctuations at flow velocity minima, suppressed by hydrodynamic interactions during periods of the flow velocity peaks. It explains evident absence of the equilibrium position at the channel axis (Fig 3A).

### Mechanical properties of hydrogel nanofilaments

Based on the length-to-diameter ratio *L* *(= L/d)*, we classified our hydrogel nanofilaments into two groups, according to their characteristic mechanical properties (see Table 1). Nanofilaments characterized by a high slenderness *L* (Table 1, highlighted left column), have a typical relative flexural stiffness *A* ~ 70 and relative extensional (Hookean) stiffness *K ~ 120*. This indicates that–for our experimental conditions–hydrogel filaments are characterized by very high bending flexibility and minimum extensibility. At the same time, their small persistence length (~*10 μm*) and a low bending stiffness of *κ = 4·10*^*−26*^ *Nm*^*2*^ determine the capability of nanofilaments to undergo thermal fluctuations. Similar mechanical properties were inferred for nanofilaments with a contour length below 40 μm and *L* ~ 250 (Table 1, highlighted right column).

**Table 1:** Mechanical properties of electrospun hydrogel nanofilaments, (semi)flexible actin filaments, and DNA. *D*, *L*, *l*_*p*_, *κ*, *l*_*p*_*/L*, *A*, *K* of hydrogel nanofilaments are reported as range of values and as mean ± standard deviation.

	Hydrogel nanofilaments		Actin [50]	(λ) DNA [51]
	L ~ 500 (L ≥ 40)	L ~ 250 (L < 40)	L ~ 7·10^3^	L ~ 10^4^
*D*	109–165 nm	105–181 nm	7 nm	2 nm
	140±19 nm	128±27 nm		
*L*	40–83 μm	26–41 μm	< 50 μm	~ 20 μm
	65±17 μm	31±5 μm		
*E*	2 kPa	2 kPa	2.6 GPa	0.3–1 GPa
*l*_*p*_	3–18 μm	3–25 μm	17 μm	50 nm
	10±4.3 μm	7.5±7 μm		
*κ*	1·10^−26^–7·10^−26^ Nm^2^	1·10^−26^–10·10^−26^ Nm^2^	7·10^−26^ Nm^2^	2·10^−28^ Nm^2^
	4·10^−26^±2·10^−26^ Nm^2^	3·10^−26^±3·10^−26^ Nm^2^		
*l*_*p*_*/L*	0.08–0.22	0.09–0.79	0.34	2.5·10^−3^
	0.14±0.04	0.25±0.25		
*A*	13–124	35–161	7·10^5^	9·10^4^
	68±37	71±42		
*K*	16–343	33–369	6·10^3^	95
	106±102	145±108		

Unfortunately, there are no experimental data for flexible nanofilaments in flow reported in the literature up to now. Hence, Table 1 includes only basic parameters of the literature data for actin [50] and short DNA [51], as closest to the present experiment. The non-dimensional mechanical characteristics *A* and *K* of these two biomolecules are scaled by the parameters of the present experimental conditions (channel width *200 μm* and flow velocity *100 μm/s)*. The elevated values of both parameters obviously indicate much higher relative mechanical stiffness of these biomolecules when compared with our inextensible but highly flexible hydrogel nanofilaments (*l*_*p*_ */ L* << 1). The shape deformations observed in the experiment resemble the behavior of long biopolymers such as actin filaments and DNA chains. However, the much higher relative flexural *A* and extensional *K* stiffness of these biomolecules should be emphasized (Table 1). This indicates that, at a similar flow configuration, the expected deformations of actin and DNA filaments would be much smaller.

### Nanofilament shape variation

To understand how the oscillatory flow affects the conformation of hydrogel nanofilaments, we followed their motion over time and evaluated the changes in their shapes as a function of time. We observed that the distribution of filaments across the whole geometry was rather uniform (S2 Fig). Although, for the present analysis we have chosen nanofilaments located in the middle part of the microchannel (area 40 μm from its centerline). This approach made it possible for us to minimize the effects of strongly varying shear rates and hydrodynamic interactions between nanofilaments and the microchannel wall.

**Fig 4:**
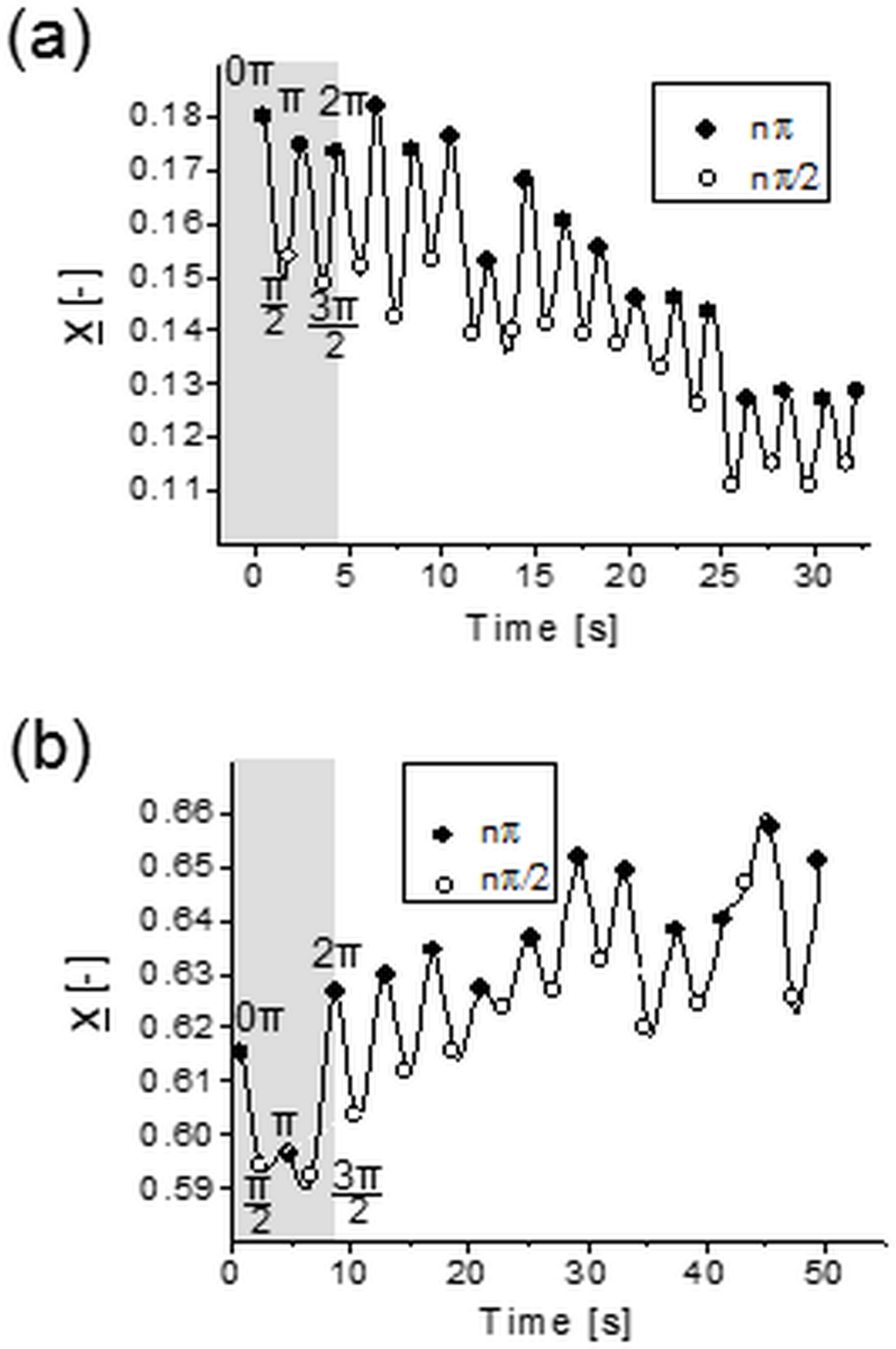
Lateral migration for hydrogel nanofilaments. (a)—toward the channel center (*d = 105 nm*, *L = 41* μ*m*, *V*_*max*_ *= 250* μ*m/s*, *relative migration velocity U*_*r*_ *= 0*.*85 10*^*−3*^; (b)—toward the wall (*d = 134 nm*, *L = 54* μ*m*, *V*_*max*_ *= 132* μ*m/s*, *U*_*r*_ *= 0*.*6 10*^*−3*^.

**Fig 5:**
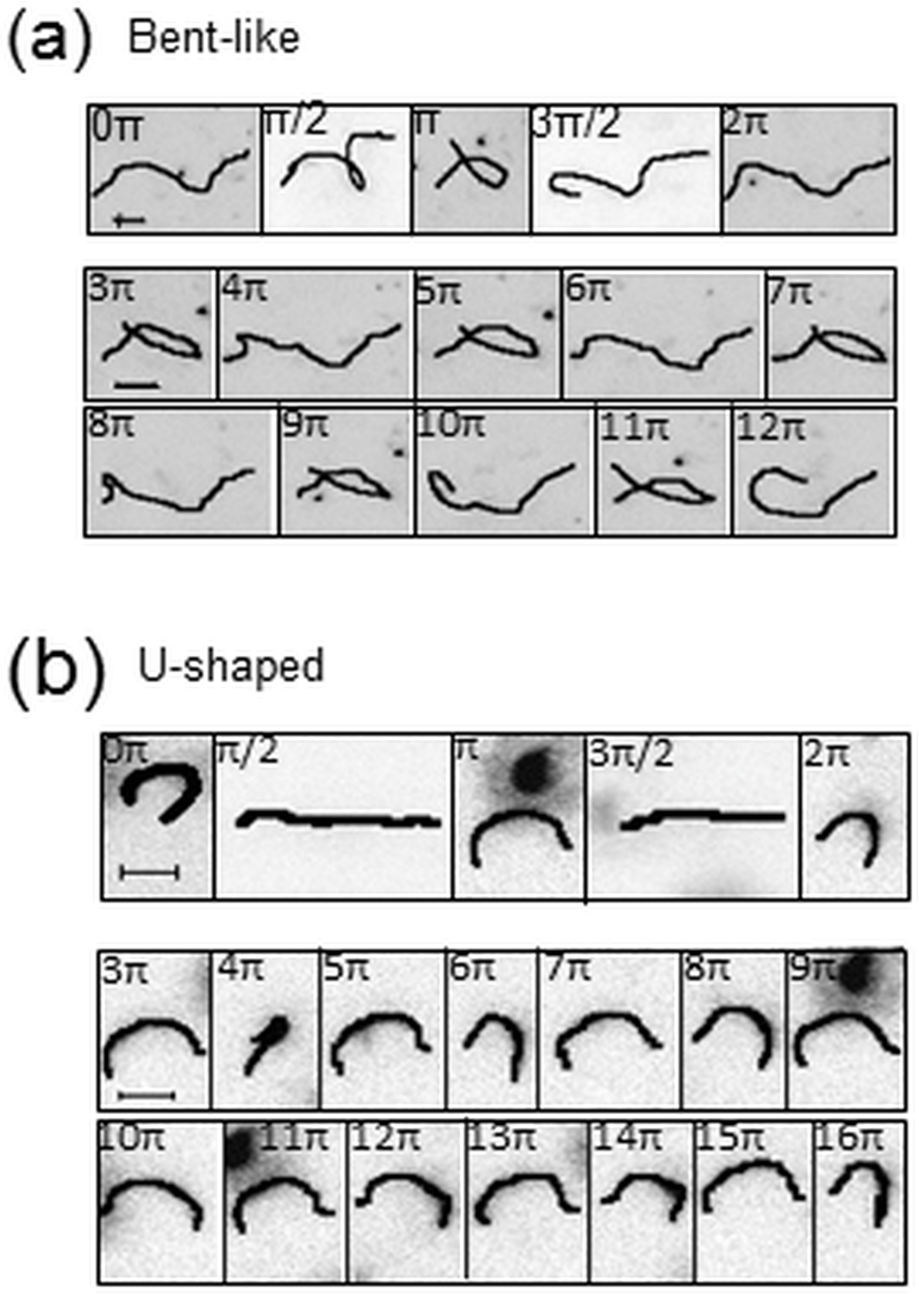
Flow induced changes of nanofilament shape observed at selected time steps during the experimental run. (a)–bent-like nanofilaments (*L* ~ *500*); (b)—short U-shaped nanofilaments (*L* ~ *250*). Scale bars denote *10* μ*m*.

Fig 4 shows two examples of trajectories of the center-of-mass position of a hydrogel nanofilament. It is noteworthy that their lateral migration is of the same order of magnitude as the one of 1 μm diameter spherical particle (comp. Fig 3). Apparently the 50 μm long deformable object (Fig 5) has “*hydrodynamic diameter”* of lateral migration comparable to that of 1 μm sphere.

**Fig 6:**
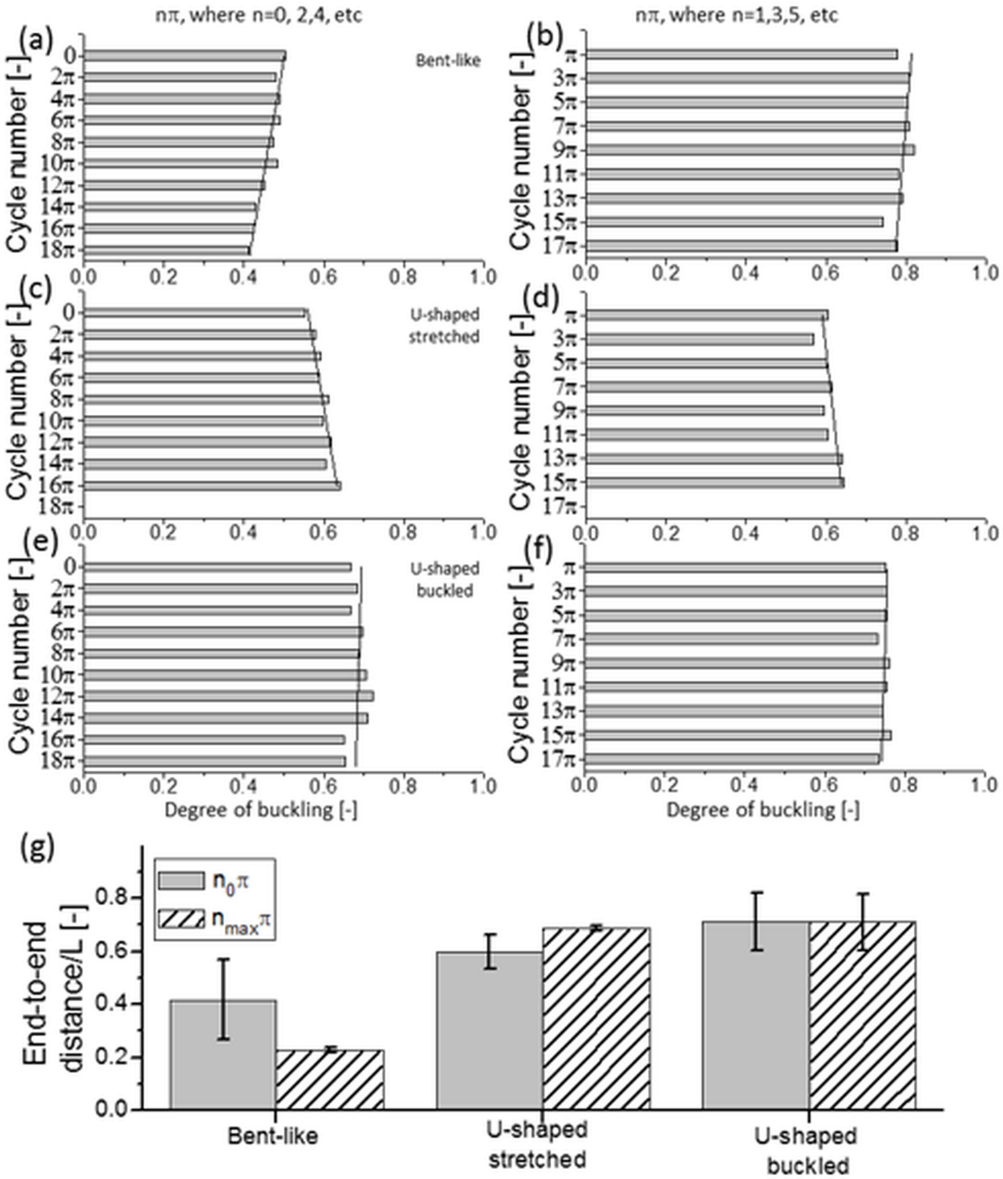
Flow induced changes in nanofilament shape observed after 9–10 oscillatory cycles. Variation of degree of buckling for bent (a, b), U-shaped stretched (c, d) and U-shaped buckled (e, f) nanofilaments recorded at each forward (*left column*) and reversed (*right column*) oscillatory cycle. Solid lines indicate the data trend. (g)–changes in the end-to-end contour length distribution for the three groups of nanofilaments, at the initial oscillatory cycle (*grey bars*) and after the final cycle (*dashed-pattern bars*).

Interesting to note are the observed differences in the filaments shape recorded at different phases of the oscillatory flow. In the group of long bent-like chains (*L* ~ 500) nanofilaments continuously cycle between uncoiled (bent) and coiled conformations (Fig 5A). The coiling of the chain always takes place during the first half of the oscillatory cycle. After the direction of the flow reverses in the second half of the cycle, the chain returns to its original uncoiled configuration. These changes in nanofilament shape are not accompanied by any sudden changes in its motility, such as tumbling or rotation. However, with an increased number of oscillatory cycles, the overall degree of chain entanglement progresses over time. Nanofilaments become more compressed at each consecutive forward (at nπ, where n is even integer) and reverse cycle (at nπ, where n is odd integer) (Fig 6A and 6B). Consistently, the total end-to-end distance decreases by a factor of two (Fig 6G left panel). These bent-like nanofilaments resemble closely the configuration of biopolymer chains in confined geometries deformed by hydrodynamic forces [26,28,33,52].

In the group of short U-shaped nanofilaments (*L* ~ 250), their initial U-shaped contour (Fig 5B) undergoes some distinct changes. During each oscillatory cycle the chain deformation shows two phases: i) a period of straightening, followed by a rotation within the *x-y* plane that results in bending in the opposite direction during odd cycles, and ii) a period of reverse straightening and rotation after which the chain goes back to its original conformation during even cycles. Over time, the chain straightens slightly at each forward and reversed cycle with respect to its initial configuration (Fig 6C and 6D), and its end-to-end distance increases slightly (Fig 6G middle panel).

Interestingly, within the group of U-shaped nanofilaments we also found a population of chains that did not undergo any changes in their elongation parameter (Fig 6E and 6F). These nanofilaments seem to maintain their U-shaped buckled deformation throughout the whole observation period (Fig 6G right panel), together with the preserved rotational movement. Importantly, regardless of their more straightened or preserved buckled configuration, both U-shaped types of nanofilaments are similar in appearance to semiflexible polymer chains conveyed in a fluid flow [24,26,27,46].

As it is shown above, hydrogel nanofilaments adopt two different shape configurations. Taking into account that the diameter and the Young modulus of bent-like and U-shaped nanofilaments are comparable, we may conclude that it is mainly their length that determines their shape deformations. In fact, all investigated configurations are characterized by relatively high Peclet number values *Pe (*= *LV*_*max*_*/D)*, relating magnitude of filaments convective transport to their longitudinal Brownian diffusion characterized by *D*. As it is shown in Table 2 Peclet number has value of approximately 7000 for bent vs. 800 for U-shaped stretched and 1600 for U-shaped buckled, respectively. It indicates small effects of translational thermal fluctuations of filaments compared to the hydrodynamic forcing. This is not true during short stop-flow time intervals in between the oscillatory cycles, where thermal fluctuations might contribute to the filament shape and position variations.

**Table 2:** Selected characteristics of hydrogel nanofilaments analyzed in the present experiment compared with the bead-spring WLC model [18–20] and the experiment with polymer fibers [18]. *Sp*, *Pe*, *K*, *A*, *U*_*r*_, *U*_*s*_ of hydrogel nanofilaments are reported as range of values and as mean ± standard deviation.

	Hydrogel nanofilaments			Bead-spring model [18–20]	Polymer fibers [18]	
	Bent-like	U-shaped buckled	U-shaped stretched		PCL	PLLA
	L ~ 500 (L ≥ 40)	L ~ 200 (L < 40)	L ~ 250 (L < 40)	L = 20	L ~ 50	L ~ 50
*Sp*	16–674	3–10	1–20	2.4·10^3^	3·10^−8^	2·10^−8^
	160±224	6±4	9±8			
*Pe*	2080–23056	1498–1844	485–1240	-	22	22
	7179±7518	1644±179	806±316			
*K*	16–343	33–114	77–369	10	2·10^7^	5·10^7^
	106±102	85±45	191±125			
*A*	13–124	35–54	62–161	0.5	7·10^7^	2·10^8^
	68±37	45±9	91±48			
*U*_*r*_	5·10^−6^–1·10^−3^	2·10^−5^–1·10^−3^	7·10^−5^–2·10^−3^	5·10^−4^ ^a^	-	-
	4·10^−4^±5·10^−4^	6·10^−4^±5·10^−4^	6·10^−4^±8·10^−4^			
*U*_*s*_	0.7–1	0.93–0.96	0.82–0.99	0.999 ^a^	-	-
	0.91±0.07	0.95±0.02	0.90±0.07			

^a^ mean value for initial fiber position *x* *= 0*.*6* [18].

At the same time, a similar persistence length and bending stiffness of hydrogel nanofilaments rule out the influence of different mechanical properties on the chain shape. This is manifested by high values of Sperm number [53] *Sp (=* 32*πηV*_*max*_*L*^4^/(*EW*)*)*. It has value approximately *160* for the bent filaments vs. *9* for U-shaped stretched and *6* for U-shaped buckled, respectively, suggesting a dominant role of flow-induced viscous stresses on nanofilament shape configurations.

**Fig 7:**
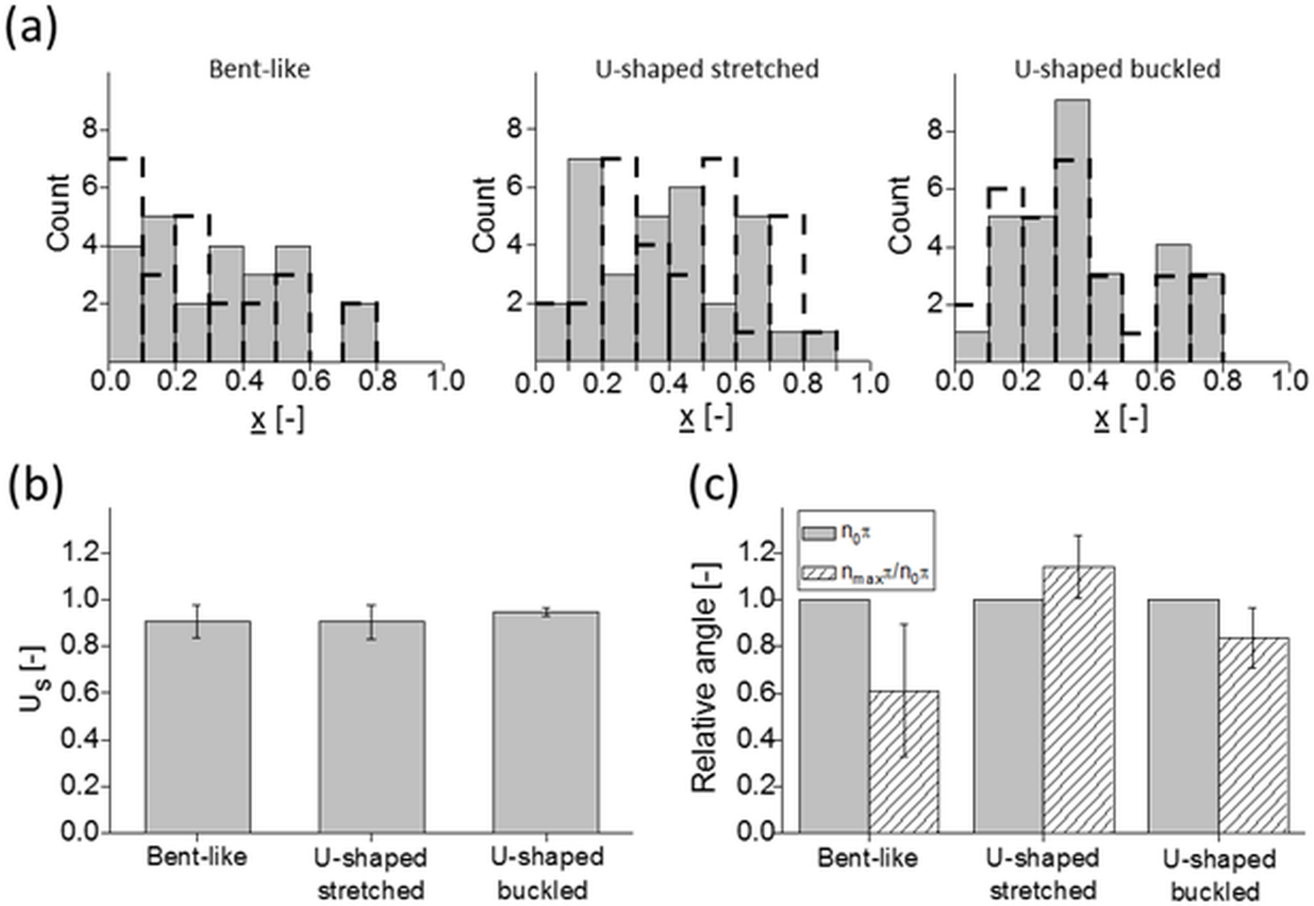
Statistics of cross-stream migration. (a)—distribution of hydrogel nanofilaments across the microchannel between centerline (0) and wall (1) at initial (*grey bars*) and final (*dashed contour lines*) oscillatory cycle; (b)—relative filament slip velocity *Us* for the three groups; (c)—relative change of the inclination angle for each group of nanofilaments: final orientation (*dashed-patterned bars*) normalized to the initial orientation (*grey bars*).

Chain conformation is one of the most important parameters affecting the diffusivity and cross-stream migration of long polymers. Therefore, to investigate how the length of filaments determines their lateral migration inside the microchannel, we followed the distribution of the center-of-mass position for bent and U-shaped filaments over time. Both types of nanofilaments show in-phase variation of their center-of-mass position along the direction of the flow applied (Fig 2A). Their initial distribution across the width of microchannel seems to be more or less uniform for each nanofilament type (Fig 7A, grey bars) thus enabling us to neglect channel entry disturbances. The application of an oscillatory flow appears to shift this distribution closer to the microchannel centerline for the bent nanofilaments, but further away for the U-shaped stretched nanofilaments (Fig 7A, black-dotted lines, left and middle panels). This happens at a comparable magnitude of the applied flow velocity (*V*_*max*_ *= 125 μm/s*), thus suggesting that the shape of filaments determines the preferable direction of lateral migration.

### Cross-stream migration of hydrogel nanofilaments

Bent-like nanofilaments migrate toward the centerline of the microchannel, while the U-shaped stretched nanofilaments migrate toward the wall. Interestingly, the relative filaments slip velocity *U*_*s*_ for these two types of nanofilaments remains similar (Fig 7B), thus suggesting that the type of nanofilament deformation does not affect their velocity lag. Furthermore, the relative filaments slip velocity *U*_*s*_ does not differ between both types of U-shaped filaments (Fig 7B). This is despite a doubled value of the applied flow velocity (*V*_*max*_ = 125 μm/s for U-shaped stretched and 250 μm/s for U-shaped buckled, respectively), and slightly increased elongation (Fig 6G middle and right). However, a higher magnitude of the flow velocity applied facilitates the migration toward the microchannel centerline, as can be seen in the case of the U-shaped buckled nanofilaments (Fig 7A, right panel). Lower flow velocity magnitude, on the other hand, causes migration toward the wall, as in the case of the U-shaped stretched nanofilaments (Fig 7A, middle panel). Therefore, the amplitude of the oscillating flow velocity applied determines the direction of migration within the group of nanofilaments with a similar shape, even though it does not affect magnitude of their migration velocity. Moreover, it also influences the orientation of nanofilaments with respect to the flow direction. Throughout the experiment, the U-shaped stretched nanofilaments diverge from alignment along the flow, while the orientation angle of the U-shaped buckled nanofilaments increases over time (Fig 7C). The bent-like nanofilaments align as well along the flow, even though–in their case—the magnitude of the applied flow velocity is lower compared to the U-shaped buckled nanofilaments.

## Discussion

As demonstrated here, a hydrogel can be electrospun into inextensible but highly deformable nanofilaments having a Young modulus as low as 2 kPa and a persistence length of around a few μm. When suspended in a viscous fluid, hydrogel nanofilaments show some bending modes which resemble the contour conformations observed for biopolymers, such as actin filaments or DNA chains [33,35]. Long hydrogel nanofilaments (*L* *~ 500*) show some transverse bends along their length, while short hydrogel nanofilaments (*L* *~ 250*) remain buckled into the U-shaped contour. Regardless of these differences, hydrogel nanofilaments stay prone to thermal fluctuation and undulate transversely or wiggle their ends freely in the solution (S5 File) [41]. This effect changes for very long filaments where–due to thermal fluctuations–some more significant conformational changes are observed, including those typical for long biomolecule knot formation (S5 File). This is possible thanks to their low bending stiffness, which is driven by Young modulus three orders of magnitude lower than that of semiflexible polymers. The high water content ensures the high flexibility of hydrogel nanofilaments, despite a ~20-times larger diameter which, in theory, should imply a more rigid structure. Additionally, the simple isotropic structure of hydrogel nanofilaments–which is in significant contrast with the complex supramolecular structure of biopolymers–adds to this flexibility by eliminating repulsive electrostatic interactions between monomers [54]. Moreover, lack of significant molecular interactions seemingly is responsible for their very low *“shape memory”*. In absence of hydrodynamic interactions once deformed filament preserves its shape for a long time, slightly modified by Brownian fluctuations only (S5 File). On other hand, in the presence of an oscillatory flow, the thermal bending fluctuations of hydrogel nanofilaments are minor (*Pe >>1*) and their conformations undergo some periodic coiling/uncoiling or rotations, depending on their length. As hydrodynamic forces promote the bending of an elastic chain once the viscous force overcomes the elastic force, the probability of bending is expected to be higher for more flexible polymer chains. The threshold of such a flexible bending is controlled by *Sperm number (Sp)*. The higher the *Sp*, the more bent the filament is [28,55,56]. Consistently, our bent-like hydrogel nanofilaments show a 17- to 27-times higher Sperm number than U-shaped nanofilaments (Table 2) and their coiling/uncoiling conformations are accompanied by length compression during each consecutive oscillatory cycle. Thus smaller *Sp* values for U-shaped nanofilaments indicate their more “rigid” conformation resulting from a larger *l*_*p*_*/L* ratio. Interestingly, the unique case of hydrogel nanofilaments buckled into U-shaped contour corresponds closely with the shape that actin filaments assume in a parabolic flow profile [26,28], shear flow [57], or when they are subjected to tension by myosin motors [58]. Also, microtubules (MTs) undergoing buckling due to polymerization forces or a lipid bilayer, assume such particular shape [59–61]. On the other hand, the coiling and stretching of longer hydrogel nanofilaments resemble closely the high flexibility of the DNA chain [30,33], which is necessary for its transport through nuclear pores [62] and for the formation of gene regulatory complexes [63]. Thus, even though the elastic mechanical properties of nanofilaments are in closer agreement with those of semiflexible actin filaments than DNA chains (Table 1), in the presence of hydrodynamic forces, hydrogel nanofilaments have the ability to mimic the behavior of both biopolymer types.

The elastic properties of inextensible and flexible biopolymer chains are commonly described using the worm-like chain (WLC) model [12,64]. The bead-spring chain models of the WLC has been mostly used to assess the hydrodynamic interactions of long polymer chains [17–21]. By following previously defined model parameters [18–20], the mechanical properties of a polymer chain in the presence of an external flow can be characterized by the relative flexural stiffness (*A*) and the relative extensional stiffness (*K*). We verified these model parameters for our hydrogel nanofilaments and found that their order of magnitude covers the range of these two parameters from previous computational studies (Table 2). However, they fall significantly behind the experimental values reported there for fibers produced from the elastomeric polymers polycaprolactone (PCL) and polylactic acid (PLLA) (Table 2 - right columns). It became clear that due to their low extensibility (high values of *A* and *K*), and low probability of bending (low *Sp* values), their dynamics could not follow the predictions of the applied WLC model [18]. On the other hand, our hydrogel nanofilaments offer a good validation of the assumptions of the bead-spring model. This is true at least in the range of configurational parameters where the effects of inertia and Brownian fluctuations are negligibly small. Moreover, the cross-stream migration of our hydrogel nanofilaments resembles closely the predictions of classical bead-spring models, where flexible polymers strive to reach their equilibrium position between 0.25 and 0.5 of the distance from the microchannel centerline [18–20,27]. However, it is worth noting that our hydrogel nanofilaments do not experience any sudden flipping that is postulated by the bead-spring model (comp. Fig 6 in Sadlej et al. [18]). Instead, the short U-shaped nanofilaments show rotational movements only. According to the model, the highest amplitude of these tumbling events is expected to occur in the vicinity of the microchannel wall. Throughout our experimental conditions it was absent, although the oscillatory character of the flow forcing might minimize the possibility to develop these sudden changes of the filament conformational shape.

The lateral migration of polymers is determined by the tendency of the chain to equilibrate its conformation in the presence of hydrodynamic forces. For hydrogel nanofilaments this depends on the initial shape and the magnitude of the flow applied. Bent-like nanofilaments migrate toward the centerline of the microchannel where the local shear rate is minimized and no additional deformations can influence the chain conformation. At the same time, in this position within the microchannel, the oscillatory velocity reaches its peak values of periodically changed flow directions, which may help to increase the chain alignment. In contrast, migration toward the wall would promote the stretching of bent-like nanofilaments, which is due to the increasing shear rate. Interestingly, at the same magnitude of the applied flow, the U-shaped stretched nanofilaments show migration in the opposite direction, namely toward the wall (Fig 4B). Migration toward higher gradients does not promote the alignment of the U-shaped stretched nanofilaments. Conversely, they tilt with respect to the flow direction. The increased magnitude of the applied flow enhances the alignment even though it prevents straightening, as can be seen in the case of U-shaped buckled nanofilaments. Indeed, at higher flow velocities, polymer chains tend to increase their orientation along the flow direction and to position themselves closer to the microchannel center [18]. This is consistent with the cross-stream behavior of the U-shaped buckled nanofilaments as compared to the U-shaped stretched filaments. On other hand, as pointed out by de Pablo et al. [65], in confined geometry and at high shear rates typical for experiments with DNA molecules, a weak migration towards the wall can occur. It is because the hydrodynamic migration effects of the two walls are essentially canceled, and the chain, which is stretched in the flow direction, is actually compacted in the wall-normal direction, allowing its center of mass to come closer to the wall. Hence, it seems evident that additional studies are necessary to elucidate details of complex interactions of shear stresses induced by the oscillatory flow, deformability of filaments due to the flow, thermal fluctuations, and the tendency of filaments to migrate across the channel.

## Conclusions

The flexible hydrogel nanofilaments presented here may be successfully used as a model of flexible biopolymers, as they can mimic closely their hydrodynamic response. Such hydrogel nanofilaments also offer a good validation of the essential assumptions of the bead-spring model of flexible fibers [18] and, therefore, should also be able to properly predict the behavior of inextensible polymers described by continuum mechanics models [17–21,23]. Moreover, as the rigidity of hydrogel nanofilaments can be easily manipulated by either changing electrospinning parameters or using different types of hydrogel materials [66], the electrospun nanofilaments have the potential to become a substitute for other types of biopolymers when studying their transport properties under flow. Additionally, such electrospun hydrogel nanofilaments may also serve as an archetype of flexible polymers for more systematic studies focusing on the development and optimization of microfluidic devices for biopolymer sorting and lab-on-a-chip technologies. Lastly, they can be used either for designing highly specialized biomimetic networks for drug delivery, or for targeting local tissue regeneration, which are essential aspects of life science. Numerous reports underline advantages of filamentous drug carriers in penetrating cancer cells [67,68]. Proper understanding of transport properties of flexible filaments in the context of microfluidics plays a crucial role in all these applications.

## Supporting information

S1 File Electrospinning chamber. (PDF)

S2 File Co-axial electrospinning equipment. (PDF)

S3 File Microchannel preparation. (PDF)

S4 File Appendix: Definition of symbols and non-dimensional numbers. (PDF)

S5 File Supplementary video file: Thermal fluctuation of nanofilaments. (MP4)

S1 Fig Pulsate pump. (PDF)

S2 Fig Initial distribution of filaments. (PDF)
