# Supplementary material for: Lateral migration of electrospun hydrogel nanofilaments in an oscillatory flow
Source: PLoS One. 2017 Nov 15;12(11):e0187815. doi: 10.1371/journal.pone.0187815 (PMC5687761; doi:10.1371/journal.pone.0187815)
Supplement: S1 File — (PDF) [file pone.0187815.s001.pdf]

S1. Electrospinning chamber

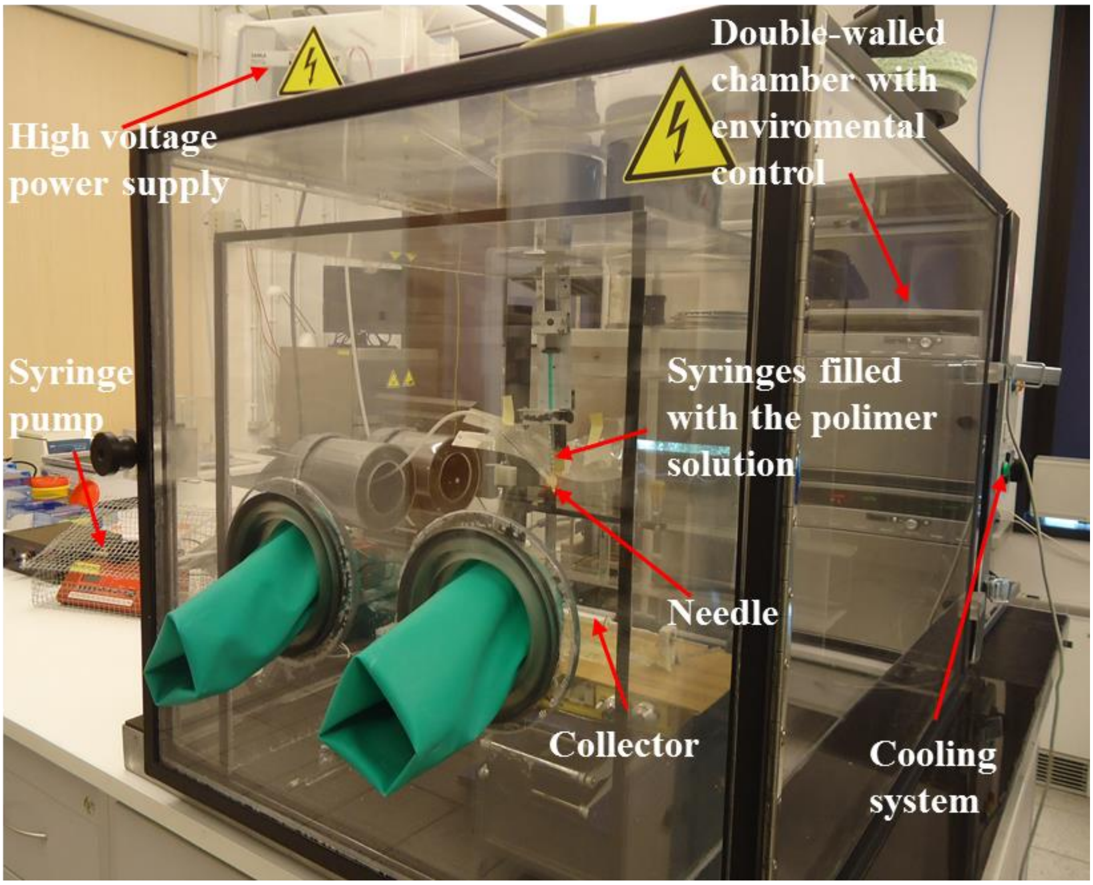

Electrospinning environmental chamber constructed at IPPT PAN

The collector is a rotating drum with a diameter of 9 mm, which rotates at a present 2000 rpm speed and moves also in a direction perpendicular to the direction of polymeric nanofibers stretching. Such a collector movement makes possible to evenly distribute fibers.

Nanofibres were electrospun using a vertical co-axial setup with the outer layer flow rate (Q_{shell}) of = 1500 µl/h. The core solution flow rate was set to Q_{core} = 1500 µl/h. Electrospinning was performed at a positive voltage of V = 15 kV. Nanofibres were collected onto a microscope slide and on the rotating drum (2000 rpm), covered with 3 cm width grounded aluminium foil. Temperature and humidity during electrospinning were T = 25°C and h = 45%, respectively.
