# Supplementary material for: Lateral migration of electrospun hydrogel nanofilaments in an oscillatory flow
Source: PLoS One. 2017 Nov 15;12(11):e0187815. doi: 10.1371/journal.pone.0187815 (PMC5687761; doi:10.1371/journal.pone.0187815)
Supplement: S2 File — (PDF) [file pone.0187815.s002.pdf]

S2. Co-axial electrospinning equipment

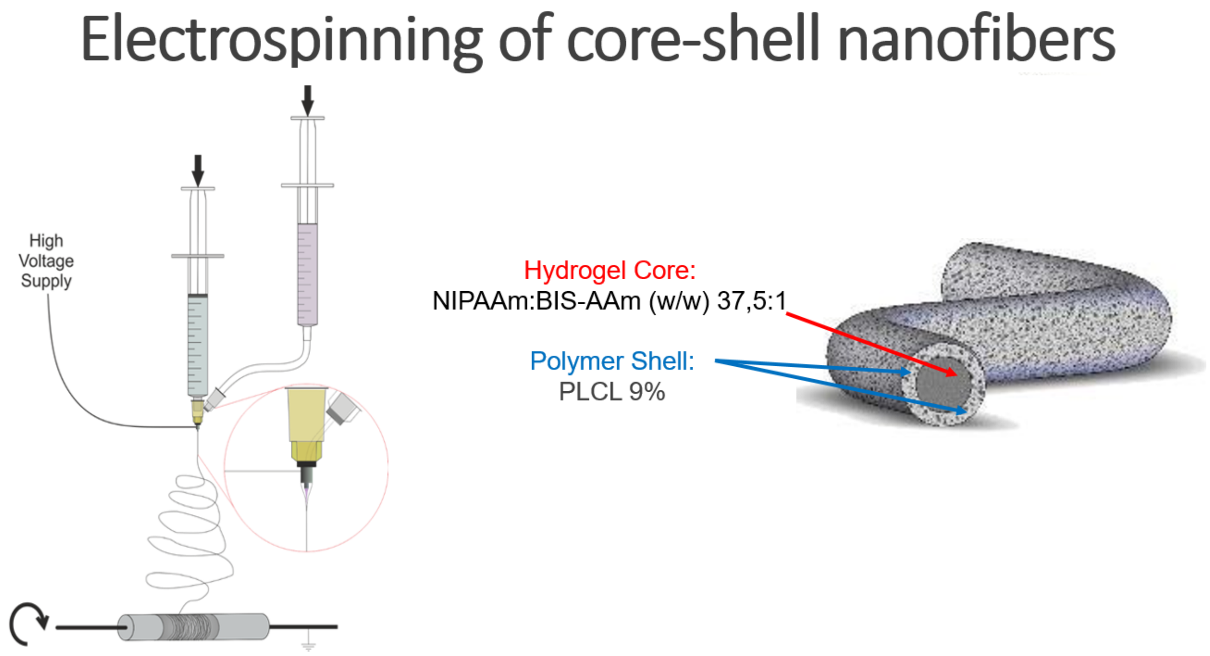

Schematic of coaxial electrospinning procedure for core-shell fibres.

In this work the core-shell electrospinning is used to fabricate hydrogel nanofilament. In our opinion it is the unique technique that allows to obtain fragile hydrogel filaments by encapsulating them in mechanically stable polymeric shell.

Two independent syringe pumps supplied polymer solutions at selected flow. Polypropylene syringes (1 ml volume) filled with mixtures of core and shell polymer respectively, were connected with a co-axial nozzle, allowing simultaneous dosing of both polymer mixtures. The handmade and disposable nozzle consisted of two needles. A 27-gauge needle was placed inside the 20-gauge needle to set the inner dimension for core-shell production.

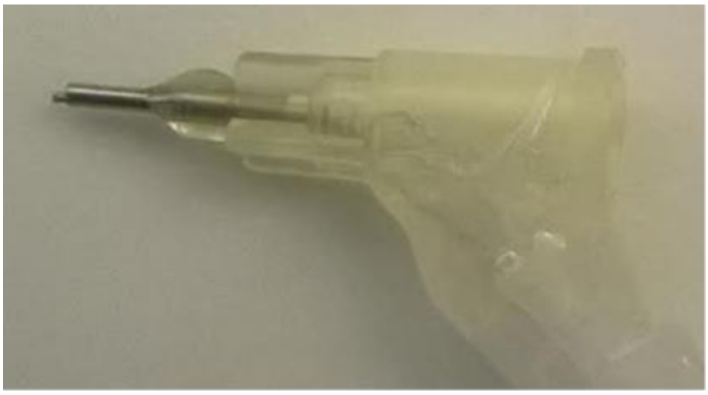

Disposable nozzle for core-shell electrospinning, designed and manufactured at IPPT PAN.
