# Supplementary material for: Lateral migration of electrospun hydrogel nanofilaments in an oscillatory flow
Source: PLoS One. 2017 Nov 15;12(11):e0187815. doi: 10.1371/journal.pone.0187815 (PMC5687761; doi:10.1371/journal.pone.0187815)
Supplement: S3 File — (PDF) [file pone.0187815.s003.pdf]

## S3. Microchannel preparation

The first step includes the design of the microchannel architecture by producing the mask. The mask then is used to prepare the matrix by negative lithography technique. This technique, called soft lithography, is commonly used in the fabrication of PDMS microfluidic chips. The essential materials for fabrication of the matrix are: backplate (silica wafers) and a suitable photoresist (Su-8 2075, MicroChem). Su-8 is a group of epoxy-based negative photoresist, which during exposure to UV become cross-linked. Only not-illuminated areas of photo resist are removed from silicon wafer by developer solvent. This procedure requires the use of a few laboratory equipment as a spin coater and UV lamp (DYMAX Flood Lamp 5000). Prepared matrices are used to achieve proper microchannel architecture, by stamp he thermocuring elastomer of poly(dimethylsiloxane) (PDMS, Dow Corning), which perfectly reflects the convex elements of the matrix. The fabricated PDMS microchannel is then sealed with a standard glass cover slip (joining method using oxygen plasma) used for observation of the phenomena studied under a microscope.

The final procedure is in accordance with the standard guidelines, and it was selected so that the depth of the finally obtained microchannels was equal 50-60 μm:

a) Clean silicon wafer using plasma cleaner:

- plasma process parameters:
  - ➢ pumping down pressure to 0.1 mbar
  - ➢ gas supply period:
    - process duration: 15 minutes
    - gas flow: 0,5 sccm
    - process pressure: 0.1 mbar
  - ➢ plasma process:
    - duration: 10 minutes
    - power: 100%

b) Spin-coated photoresist (the process requiring limited access to light):

- 2 ml of Su-8 supply on central part of silicon wafer;
- spin at 500 rpm for 20 seconds and next 2000 rpm for 30 seconds;
- removal of Su-8 excess from the edge bead from silicon wafer by use MicroChem's Su-8 developer (during 10 second, since 15 second before the end of spin-coated process);

- Soft Bake step 1 – leave of silicon wafer with photoresist on $65^{o}C$ hotplate during 3 minutes;
- Soft Bake step 1 – leave of silicon wafer with photoresist on $95^{o}C$ hotplate during 9 minutes;
- Soft Bake step 1 – leave of silicon wafer with photoresist on $65^{o}C$ hotplate during 1 minutes, and to cool to room temperature;
- leave silicon wafer with photo resist in room temperature and dark place during 10 minutes for stabilisation.

c) Illumination with UV light through a mask:

- to the surface of photoresist the mask, made by Computer to Film CtF technique) and long pass filter (LP360, to eliminate UV radiation below 350 nm, to obtain vertical sidewalls in the photoresist);
- illumination with UV (density of power $225mW/cm^2$) for 5 seconds;
- leave silicon wafer with photoresist in room temperature and dark place for 10 minutes for stabilisation.

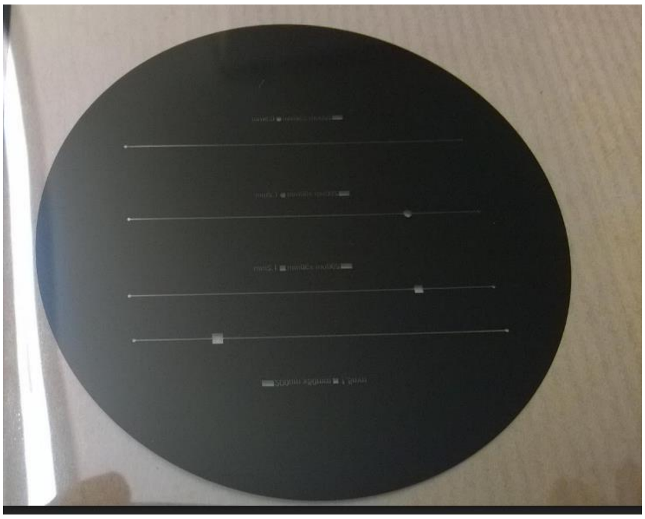

Mask – film with structure of designed microchannels. Microchannels is the transparent areas of the mask, through which the UV light during exposure is curing the Su-8.

d) Master:

- Soft Bake step 2 – leave of silicon wafer with photoresist on $65^{o}C$ hotplate during 2 minutes;
- Soft Bake step 2 – leave of silicon wafer with photoresist on $95^{o}C$ hotplate during 7 minutes;

- Soft Bake step 2 – leave of silicon wafer with photoresist on 65ºC hotplate during 1 minutes, and to cool to room temperature;
- Development - selectively remove a photoresist that has not been exposed to UV. Development time for 50-60 μm thickness of photoresist was equal 7 minutes. After this time, master should be washed with isopropanol alcohol. Absence of white blots denotes complete removal of unexposed photoresist from master. The last step was drying with air.

e) PDMS poured on the master:

- preparation of the PDMS mixture consists of mixing of two liquid components: dimethylsiloxane (monomer) and crosslinker, in proportion of 10: 1, respectively;
- after mixing the PDMS mixture must be degas under reduced pressure (gradual reduction to 15 mbar);

f) Open microstructure in PDMS:

- PDMS was pour on the master and put to the oven with temperature 60ºC for 4 hours. To obtain crosslinked polysiloxane resin.

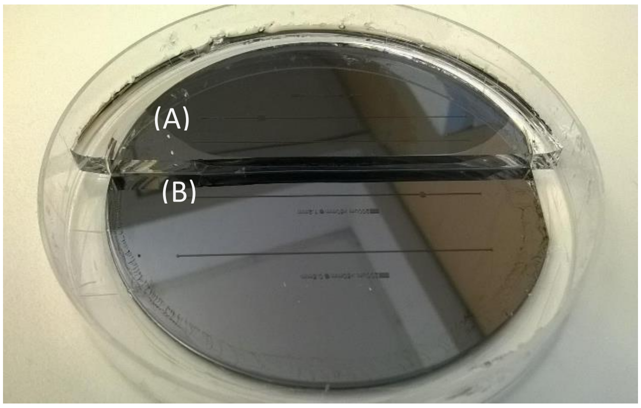

Microstructures in PDMS (A) and master (B).

g) Microfluidic device:

- PDMS is hydrophobic, but for attaching it to the glass slide it is necessary to modify its surface properties by using oxygen plasma;
- piece of PDMS with microchannels and clean glass slide are attached with use the plasma cleaner chamber;
- parameters of plasma process:
    - ➢ pumping down pressure: 0.1 mbar
    - ➢ gas supply period:

- duration: 5 minutes
- gas flow: 0.5 sccm
- process pressure: 0.1 mbar

➢ plasma process:
- duration: 1 minutes
- power: 1%

- Connect microfluidic device with steel capillaries or plastic tubes.

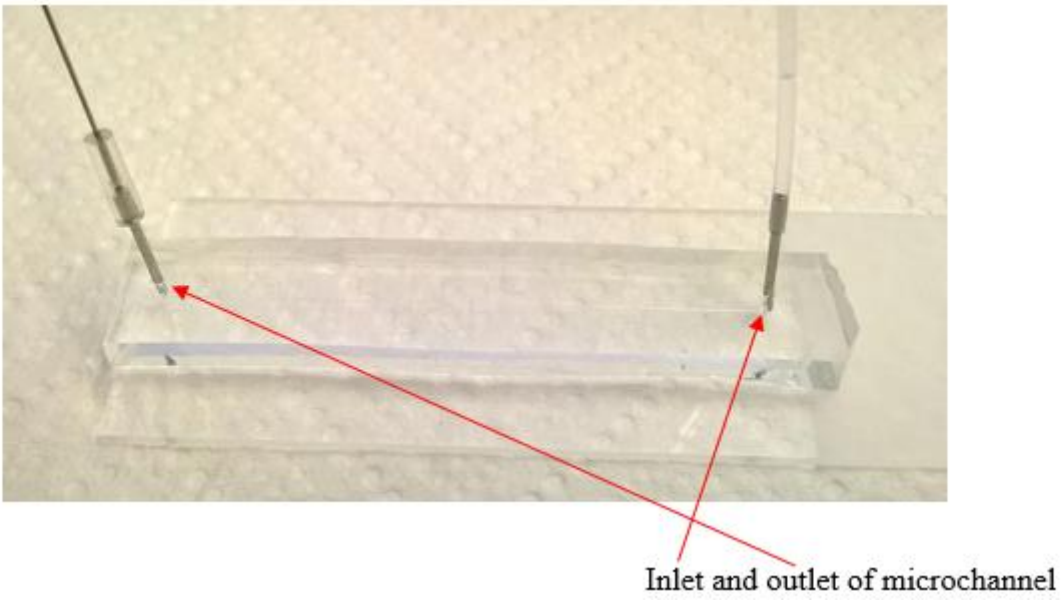


Final form of PDMS microchannel attached to glass slide.
