# Supplementary material for: Lateral migration of electrospun hydrogel nanofilaments in an oscillatory flow
Source: PLoS One. 2017 Nov 15;12(11):e0187815. doi: 10.1371/journal.pone.0187815 (PMC5687761; doi:10.1371/journal.pone.0187815)
Supplement: S4 File — (PDF) [file pone.0187815.s004.pdf]

S4 File. Appendix

1. Selected symbols characterizing experiment and materials

k_B - Boltzmann's constant [kg m^2/s^2 K]
l_p – persistence length [m]
d – particle (filament) diameter [m]
x - distance from the channel center line [m]
$\underline{x}$ – relative distance from the channel center line [-]
D - diffusion coefficients [m^2/s]
E – flexural Young modulus of filaments [Pa]
E_x – extensional Young modulus of filaments [Pa]
W – channel width [m]
L – filaments contour length [m]
T - absolute ambient temperature [K]
U_r – absolute relative migration velocity, ratio of averaged over observation time lateral velocity of particle (filament) to the maximum flow velocity V_{max} [-]
U_s – absolute slip velocity, ratio of translational velocity of particle (filament) to the calculated local fluid flow velocity [-]
Q – flow rate through the channel [m^3/s]
$V(x,y,z,t)$ – flow velocity of liquid in the channel [m/s]
V_{max} – maximum absolute flow velocity at the channel axis [m/s]
η - dynamic viscosity of the carrier fluid [Pa s]
ρ - density of the carrier fluid [kg/m^3]
ω - angular oscillation frequency of the flow [rad/s]
γ – shear rate for the channel flow [1/s]

2. Non-dimensional parameters characterizing filaments and flow

Reynolds number (*Re*)

It is the ratio of inertial forces to viscous forces in fluid flow. Small Reynolds number ($Re \ll 1$) justifies to reduce complex, nonlinear description of fluid flow to simple linear relation of force and viscous friction, called Stokesian approximation. For the channel flow the Reynolds number based on its width ***W*** is given by:

$$Re = V_{max} * W * \rho / \eta ,$$

Womersley number (*Wo*)

The Womersley number expresses ratio of the oscillatory inertia force to the viscous damping force for pulsating channel flow. It is defined as:

$$Wo = W * \mathrm{sqrt}(\rho * \omega / \eta).$$

In the present study Womersley numbers ($Wo < 1$), the effect of flow pulsations is sufficiently low and a parabolic velocity profile develops during each cycle. At high Womersley numbers flow velocity close to the walls lags the fluid flow at the channel center, and the velocity profile is characterized by inflections visible close to the wall.

Strouhal number (*St*)

The Strouhal number for the oscillating flow describes ratio of the squared Womersley number to the Reynolds number:

$$St = Wo^2 / Re = \omega * W / V_{max}$$

For large Strouhal number ($St > 1$) viscous effects dominate flow and the parabolic velocity profile develops in the channel at each pulsation phase.

Peclet number (*Pe*)

The Peclet number describes ratio of advective to diffusion driven transport rates for a particle carried by the fluid flow. It is defined as:

$$Pe = L * V_{max} / D$$

Large Peclet number ($Pe \gg 1$) indicates flow-induced longitudinal translation transport dominating Brownian diffusion.

Sperm number (*Sp*)

The Sperm number characterizes the relative magnitudes of viscous and elastic forces and can be defined as:

$$Sp = 32*\pi *\eta * V_{max} * (L/d)^4 / E*W$$

For *Sp* >> 1 the viscous forces dominate elastic forces and a flexible filament easily deforms according to local shear stresses.

Flexural stiffness (*A*)

It describes ratio of bending stiffness of the filament related to the effected hydrodynamic force of ambient flow amplitude. A large value of ***A*** indicates stiffer (less flexible) filament.. The flexural stiffness is defined as:

$$A = E * d / 32* \eta *V_{max},$$

Extensional stiffness (*K*)

It describes ratio of the extensional filaments deformation force related to a hydrodynamic force of the ambient flow amplitude. It is defined as:

$$K = E_x * d^2 / (4*L*\eta *V_{max})$$

Extensional stiffness is defined in terms of extensional Young modulus E_x. For the filaments material E_x was obtained from separately measured Hookean spring constant.
