# Supplementary material for: Lateral migration of electrospun hydrogel nanofilaments in an oscillatory flow
Source: PLoS One. 2017 Nov 15;12(11):e0187815. doi: 10.1371/journal.pone.0187815 (PMC5687761; doi:10.1371/journal.pone.0187815)
Supplement: S1 Fig — (PDF) [file pone.0187815.s006.pdf]

S1 Fig. Pulsate pump

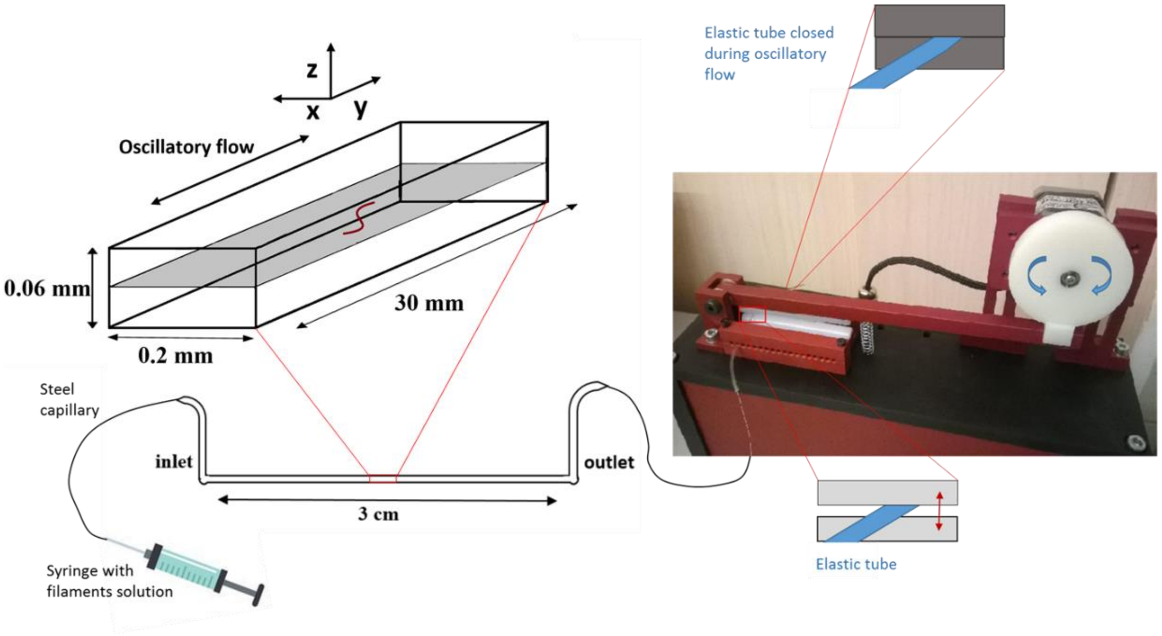

Computer controlled pulsating pump based on periodic squeezing of elastic tube by rotating wheel. Own construction by K. Z.
