# Supplementary material for: Lateral migration of electrospun hydrogel nanofilaments in an oscillatory flow
Source: PLoS One. 2017 Nov 15;12(11):e0187815. doi: 10.1371/journal.pone.0187815 (PMC5687761; doi:10.1371/journal.pone.0187815)
Supplement: S2 Fig — (PDF) [file pone.0187815.s007.pdf]

S2 Fig. Initial distribution of nanofilaments

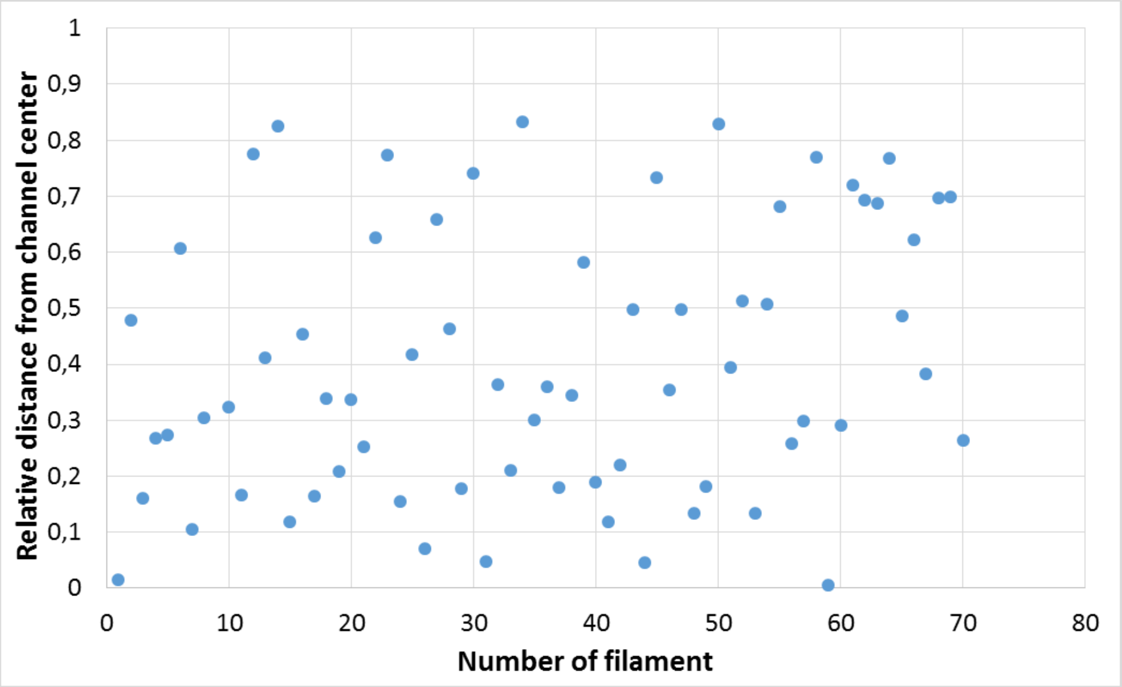

Observed distribution of investigated nanofilaments across the whole plane of observation was rather uniform, except wall region. Statistics obtained from the first images of 35 experimental sequences.
